# Co-development of a training programme on disability for healthcare workers in Uganda

**DOI:** 10.1186/s12913-024-10918-z

**Published:** 2024-04-03

**Authors:** Tracey Smythe, Andrew Sentoogo Ssemata, Sande Slivesteri, Femke Bannink Mbazzi, Hannah Kuper

**Affiliations:** 1 International Centre for Evidence in Disability, London School of Hygiene & Tropical Medicine, London, UK; 2 Division of Physiotherapy, Department of Health and Rehabilitation Sciences, Stellenbosch University, Cape Town, South Africa; 3 Medical Research Council/Uganda Virus Research Institute and London School of Hygiene & Tropical Medicine Uganda Research Unit, Entebbe, Uganda; 4 Department of Global Health and Development, London School of Hygiene & Tropical Medicine, London, UK

**Keywords:** Disability, Training, Healthcare worker, Education, Low- and middle-income, Development, Uganda, MRC framework

## Abstract

**Background:**

Approximately 1.3 billion people worldwide face barriers in accessing inclusive healthcare due to disabilities, leading to worse health outcomes, particularly in low and middle-income countries (LMIC). However, there is a lack of training of healthcare workers about disability, both globally and in Uganda.

**Objectives:**

To use mixed research methods to develop a comprehensive training program with standardisedelements for healthcare workers in Uganda, focusing on improving their knowledge, attitudes, and skills inproviding care for people with disabilities.

**Methods:**

The Medical Research Council (MRC) approach was employed to guide the development of the training intervention. We conducted an umbrella review to gather relevant literature on disability training for healthcare workers. Interviews were conducted with international experts to gain insights and perspectives on the topic. Additionally, interviews were undertaken with people with disabilities and healthcare workers in Uganda to understand their experiences and needs. A participatory workshop was organised involving key stakeholders, to collaboratively design the training material based on the findings from these data sources.

**Results:**

Eight review articles examined training programs for healthcare workers on disability. Training settings ranged from specialised clinical settings to non-clinical settings, and the duration and evaluation methods of the training varied widely. Lectures and didactic methods were commonly used, often combined with other approaches such as case studies and simulations. The impact of the training was assessed through healthcare worker reports on attitudes, knowledge, and self-efficacy. Interviews emphasised the importance of involving people with disabilities in the training and improving communication and understanding between healthcare providers and people with disabilities. Five themes for a training on disability for healthcare workers were generated through the workshop, including responsibilities and rights, communication, informed consent, accommodation, and referral and connection, which were used to guide the development of the curriculum, training materials and training approach.

**Conclusion:**

This study presents a novel approach to develop a training program that aims to enhance healthcare services for people with disabilities in Uganda. The findings offer practical insights for the development of similar programs in LMICs. The effectiveness of the training program will be evaluated through a pilot test, and policy support is crucial for its successful implementation at scale.

**Supplementary Information:**

The online version contains supplementary material available at 10.1186/s12913-024-10918-z.

## Key messages

1. Healthcare workers require training to effectively address the health concerns of people with disabilities, yet this is rarely included in curricula worldwide.

2. Uganda recognises the importance of addressing this issue and is taking steps to improve training programs about disability for healthcare workers.

3. We used multiple research methods (umbrella review, semi-structured interviews, participatory research and collaborative design) to co-develop a comprehensive training program with standardised elements for healthcare workers in Uganda, focusing on improving their knowledge, attitudes, and skills in providing care for people with disabilities.

4. The developed training material could be adapted for healthcare workers in other resource-limited settings, and policy support is needed to ensure its implementation at scale.

## Introduction

Approximately 16% of the world’s population, or 1.3 billion people, live with a disability, and the majority live in low and middle income countries (LMIC) [1]. Access to inclusive healthcare services is vital for promoting health equity and social inclusion for all [2, 3]. However, healthcare workers’ lack of knowledge, skills, and attitudes towards disability remains a significant barrier to achieving this goal [4]. Efforts to integrate disability-related education and training into healthcare curricula and continuing education programs, as emphasized by the WHO report [1], are crucial in addressing this issue. Equipping healthcare workers with the necessary tools and resources to provide effective, respectful, and culturally sensitive care to people with disabilities is essential in meeting their needs and promoting positive health outcomes. People with disabilities want to be “expected, accepted and connected” by the health system [2]. This requires training to ensure that healthcare staff are aware of disabilities, possess relevant skills, and maintain positive attitudes, enabling them to make appropriate linkages to other necessary care. Efforts to integrate disability-related education and training into healthcare curricula and continuing education programs are crucial in addressing this issue [1–3, 5].

Training on disability can lead to improvements in the knowledge and attitudes of healthcare workers towards people with disabilities throughout all stages of their careers. For example, two medical colleges in the US integrated disability across medical student training curricula, and medical students improved their knowledge, attitudes, and core competencies in treating patients with disabilities [6]. In Rwanda, continuing professional development training about childhood disability used case studies and clinic visits, and instructors gave participants’ immediate feedback on their practice. As a result, participants demonstrated improved clinical decision making skills in paediatric rehabilitation [7]. Similarly, programmes that invited people with disabilities as teachers found that participants believed the nonclinical interaction enhanced their comfort and attitudes towards people with disabilities [8, 9].

Despite the global recognition that well-trained, fairly distributed and motivated healthcare workers are critical to improving population health and achieving universal health coverage (UHC) and Sustainable Development Goals (SDGs) [10], there are limited standardised training programs focusing on disability [11], especially in LMICs. Existing programs cover diverse content areas such as general disability awareness, condition-specific knowledge, rehabilitation skills, assistive technology, inclusive design, universal design for learning, mental health, and primary healthcare (Table 1). There has been little consideration to date on what is optimal in terms of content of disability training for healthcare workers.

**Table 1:** Examples of training programmes delivered to healthcare workers on disability

Topic	Training Design	Examples
General disability awareness and sensitivity training	Designed to increase understanding of the various types of disabilities and the unique needs of people with disabilities. It may also cover topics such as disability rights and laws, and strategies for communication and inclusion.	Ministry of Health (MoH) and Clinton Health Access Initiative (CHAI) (Zambia): 5 day training course targeting nurses, MoH staff.
Condition-specific training	Focused on a particular condition, such as cerebral palsy or autism. It can provide detailed information on the causes, symptoms, and treatment options, as well as strategies for working with people who have that condition.	Latika Roy Foundation, Sightsavers (India): Training on an assessment tool for developmental disabilities targeting community healthcare workers
Rehabilitation skills training	Designed to teach healthcare workers the skills they need to provide rehabilitation services to people with disabilities. This may include training in areas such as physical therapy, occupational therapy, or speech therapy.	Comprehensive Community Based Rehabilitation (CCBRT, Tanzania): 2 day training course including didactic and workshops targeting healthcare workers and development NGOs
Training on assistive technology	Designed to provide healthcare workers with skills to work with, and provide information about, assistive technology.	Light for the world (Mozambique): Training on eye health ICRC (Ethiopia): Training on prosthetic and orthotic technology
Inclusive design and Universal Design for Learning Training	Designed to provide training on creating environments and materials that are inclusive to everyone, with specific focus on people with disabilities	Plan International: community awareness raising on disability that has been used to train village healthcare workers
Mental Health and Disability training	Focused on understanding mental health conditions and disabilities and the intersection of both, providing the healthcare worker with the necessary knowledge to understand, identify and support those with both conditions.	National Health Services (NHS, UK): 2 full day training sessions (blended learning) targeting health and social care workers Strong Minds and TPO (Uganda): mental health trainings to healthcare workers
Primary health care for people with disabilities training	Designed to enable primary healthcare workers to deliver disability-inclusive primary health care	MoH and UNFPA (Ecuador): Handbook on sexual and reproductive health for all disabilities

There are various potential approaches to training, including face-to-face or remote, involvement of people with disabilities or not, and emphasis on knowledge, skills, or attitudes, as well as different underlying models, such as medical or rights-based [11, 12]. Despite this range of possibility, there is limited collation or scrutiny of information, including input from people with disabilities and healthcare workers, to identify the most effective approach. Additionally, LMICs face unique challenges in healthcare provision, including resource constraints and limited access to education and training [13], which may contribute to the acute lack of training opportunities for healthcare workers. For instance, Uganda encounters significant challenges in training healthcare workers on disability due to limited resources, infrastructure, and cultural barriers that may stigmatise or exclude people with disabilities [14–16].

The objective of this study was therefore to use multiple research methods to develop a comprehensive training program with standardised elements for healthcare workers in Uganda, focusing on improving their knowledge, attitudes, and skills in providing care for people with disabilities. This paper describes the development of the disability training and presents the framework of the training material developed. We provide an in-depth examination of the training needs of healthcare workers in the area of disability and offer a practical solution in the form of the developed training material that can be used to improve the knowledge and skills of healthcare workers on disability. A future study will pilot test and evaluate the training material.

## Methods

We used the Medical Research Council (MRC) approach, which involves a systematic and evidence-based framework to development of a programme [17]. It emphasises the importance of involving stakeholders throughout the process and ensures that interventions are evidence-based, feasible, and acceptable to those involved in their implementation. This approach aims to create effective and sustainable interventions that improve health outcomes and services.

### Overview of methods

This study utilised data from an umbrella review, interviews with international experts on disability training for healthcare workers, and interviews with people with disabilities and healthcare workers in Uganda. The data informed the design of a workshop that involved key stakeholders, including healthcare workers and people with disabilities, in co-creating the training material. The training material was developed based on information gathered from the umbrella review, interviews, and design workshop.

### Umbrella review

The umbrella review was conducted to identify systematic reviews and meta-analyses of studies that examined associations between training of healthcare workers on any disability and change in healthcare worker behaviour, attitude or treatment delivered. The protocol for this study was registered in the International Prospective Register of Systematic Reviews (PROSPERO), reference number #CRD42023390881. We used the Preferred Reporting Items for Overviews of Reviews (PRIOR) statement for conducting umbrella reviews. We searched PubMed for studies published in English between 1st January 2015 and 11th January 2023, using the terms (“train*” OR “educat*”) AND (“healthcare worker” OR “health professional” OR “medical professional”) AND (“disability” OR “impairment”) filtered for systematic reviews and meta-analyses for this rapid umbrella review. Inclusion criteria were established as: systematic reviews or meta-analyses that evaluated training on disability (intervention) for any health professional (population). Studies were excluded if published prior to 2015 to ensure the currency and relevance of information, and if in any other language than English. We searched reference lists of included studies for additional eligible papers.

Two reviewers (TS, ASS) independently assessed study eligibility and extracted the data. The risk of bias of the included studies was assessed using AMSTAR 2 (A MeaSurement Tool to Assess systematic Reviews) [18], designed to appraise systematic reviews that include randomised controlled trials. The instrument provides a broad assessment of quality, including flaws that may have arisen through poor conduct of the review with uncertain impact on findings. We developed and pilot-tested an extraction tool in Excel, to systematically record information from included studies. Extracted information included: (1) Publication characteristics: author, title, year of publication, setting/country; (2) Study design: study design, sample size; (3) Participant characteristics: cadre, and any other relevant descriptive data; (4) Outcomes: effect size of training. Data were extracted by TS and checked for accuracy by ASS. Where studies included information on training other professionals (e.g. police officers, teachers) only data on healthcare workers were extracted. Data were also only extracted on training when reviews included additional information (e.g. use of disability measurement tools). We narratively synthesised the results.

### Semi-structured interviews

Interviews were conducted with international experts with experience of delivering training on disability in January 2022, in order to explore current practices and to identify gaps in practice and policy. Six experts were purposively sampled to represent people with and without disabilities, in low and high income settings. They were interviewed by TS, a physiotherapist and epidemiologist from Zimbabwe with mixed-methods expertise. Interviews were held online using Zoom. Verbal informed consent was given. A set of open-ended questions (Supplementary file 1) elicited detailed responses on the experiences, perspectives and practices of experts in the field of healthcare worker training on disability. The interviews were recorded and transcribed for analysis. Transcripts were coded and thematically analysed using NVIVO.

Interviews were then conducted in Uganda with people with disabilities and healthcare workers. Written consent was given. People with disabilities were asked what they wished healthcare workers would know about disability, and interviews with a range of healthcare workers were used to gather more detailed information on their specific training needs and to understand their perceptions of the current training available. Semi-structured interviews were conducted in-person with 17 healthcare workers and 27 people with disabilities in Luuka District, Uganda. ASS and SS, Ugandan social scientists with expertise in qualitative methodology undertook the interviews. Participants were recruited through existing networks, non-government organisations and health facilities. The healthcare workers were selected from the eight sub-counties that make up Luuka district based on cadre (clinical officers, midwives, nurses, village health trainers, laboratory technicians, health officers), and level of health facility (health centre, II, III or IV). The people with disabilities were purposively selected to ensure representation of age, gender and impairment. Interviews were undertaken with persons with visual impairment (*n* = 5), physical impairment (*n* = 5), multiple impairment (*n* = 5), cognitive/ intellectual impairment (*n* = 5) and albinism (*n* = 1). Interviews with people with hearing impairment (*n* = 6) were conducted by a member of the research team who is deaf (Supplementary file 2: Participant demographics). The semi-structured interviews explored experiences and perspectives of delivering and receiving health services (Supplementary file 3: Healthcare worker, Supplementary file 4: People with disability). The interviews were conducted in a private and comfortable setting and lasted 50–80 min. They were audio recorded and transcribed. Those conducted in Lusoga and Luganda were translated to English. The transcripts were coded and manually analysed using a deductive thematic analysis approach.

### Design workshop

A design workshop was held to develop a framework for the disability training intervention in Entebbe Uganda in September 2022. The workshop brought together a group of people with diverse disabilities (*n* = 7) and healthcare workers and medical educationalist (*n* = 5) to actively participate in the design process over 2 days. The workshop was facilitated by TS and ASS. The workshop consisted of a series of participatory activities, including group discussions, brainstorming sessions, and small group exercises. These activities were designed to elicit the perspectives, experiences, and recommendations of participants. The workshop concluded with a consensus-building activity, where participants discussed and agreed on the key components of the training framework.

### Development of training material

A theory of change was used to inform the content design and implementation of the training framework. The research team created the first theory of change model, led by TS, drawing on information gathered from the international experts. The theory of change approach involved identifying the desired outcomes of the training and the necessary steps and activities to be put in place to achieve those outcomes. The training material was then developed by the study team drawing on evidence from the umbrella review, interviews and design workshop on the most effective training strategies for healthcare workers on disability, and was refined based on feedback from five people with disabilities.

## Results

**Fig. 1:**
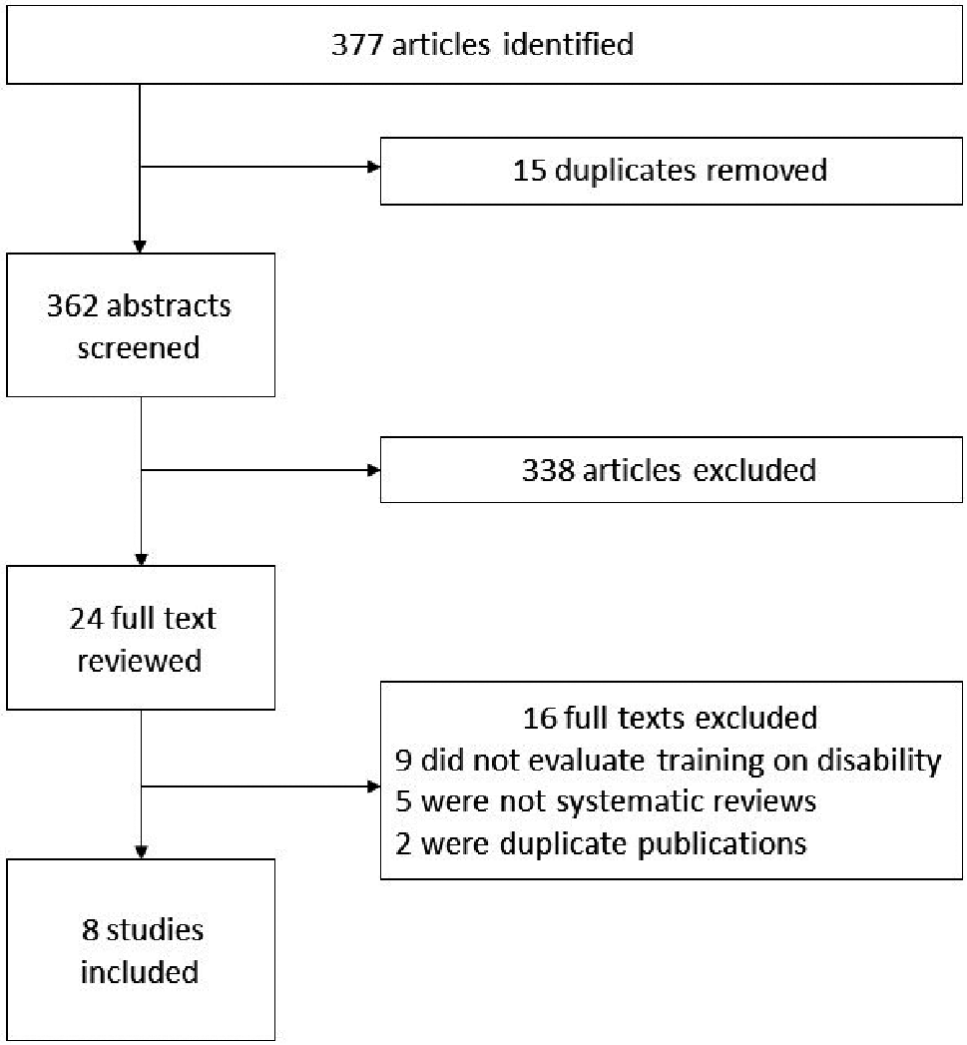
PRISMA for umbrella review of systematic reviews and meta-analyses on training of healthcare workers on disability

The literature search for the umbrella review yielded 377 studies, with 24 full-text articles selected for further review. Eight review articles were eligible for inclusion in the final analysis (Fig. 1). A total of 227 studies were included in the reviews, but only 4 studies were conducted in LMICs. The reviews related to training in intellectual and developmental disabilities, mental health, and all disabilities. Three systematic reviews were rated with high confidence on the AMSTAR2 tool, two rated with medium and three rated with low confidence (Supplementary file 5). The systematic reviews receiving a low confidence rating in their findings were evaluated as such because they failed to pre-register the review and did not include an appropriate evaluation of bias, including publication bias of the included studies.

The training settings included specialised clinical settings, non-specialised clinical settings (inpatient and outpatient), continuity-clinic based, non-clinical settings, medical schools, GP practices, primary care clinics, and various other settings such as clinics, camps, schools, residential settings, and universities. The timeline of training and training evaluation methods varied across the articles; Some articles reported single-session training, short-term training (< 1 month), and longer-term training (1–3 months or > 3 months).

Lectures and didactic (instructional) methods were the most commonly reported teaching methods in disability education, often combined with other approaches, such as case studies, simulations, and placements. Some training programmes leverage multimedia tools, such as video recording or simulations, to enhance the learning experience. The content of the education typically includes disability from a rights-based perspective, as well as particular skills, such as sign language for medical and pharmacology terms (Table 2). Many programmes involved people with disabilities as active participants in the education, such as simulating patients or serving as teachers to help run activities. Through these interactions, contact with people with disabilities was transformative, leading to significant changes in attitudes and perceptions of participants.

**Table 2:** Training on disability effect estimates for healthcare workers reported in systematic reviews (2015–2022)

Primary author, Year [Ref]	Target disability	Studies N	Countries [LMICs] Country name	Target cadre (n)	Target outcome (n)	Pedagogical methodology (n)	Measurement tools/ Evaluation methodology (n)	Participants trained N	Evidence of impact	Confidence rating
Adirim Z, 2021 [27]	Intellectual and developmental disabilities	16	2 [0] USA (14) Canada (2)	Pediatrics (12) Psychiatry (3) Family Medicine (1)	Awareness (6) Knowledge (13) Other/unclear (1)	Clinical rotation: (9/16) theoretical/ pedagogical: (14/16) didactic or seminar: (12/14) clinical practice: (10/11), simulation, (1/11). Immersive experiences: (7/16) interactive approaches (5/16)	Knowledge assessment (9/16) self-reported learning: (9/16) evaluation of intervention (3/16) clinical changes (2/16) observed behavioural change (1)	Not reported	Narrative summary: improved knowledge, skills, competence, positive attitudes	Low
Booth A, 2017 [28]	Mental health	2	2 [0] USA (2)	a) Child welfare case officers b) Community practitioners	Knowledge and referral	Face-to-face, didactic and interactive training with video demonstrations of available evidence based practice	54 item questionnaire developed by study team to evaluate knowledge of mental health conditions	a) 67 b) 182	Intervention group had significantly increased awareness of evidence based practice	Medium
Cox A, 2015 [29]	Intellectual disabilities	19	Not reported	Direct care, psychologist, manager not reported (8)	1) Health outcome: Improvement in client behaviour: (9/19)	Not reported	1) Direct observation of client behaviour (8) (Combination of two study groups) 2) questionnaires (9)	Not reported	Narrative summary: trend toward change in behaviour of clients	Low
Ioerger M, 2019 [30]	All disabilities	77	12 [4] USA (36) UK (19) Canada (6) Australia (7) Brazil (1) Nigeria (1) Pakistan (1) S.Africa (2) Croatia (1) Ireland (1) Israel (1) New Zealand (1)	Medical students	Students’ disability knowledge (35), skills (28), or attitudes (35)	Lectures most common (36), followed by reflection (25) and small group discussions (25).	1) Attitude change 2) Skills acquired 3) Knowledge 4) General feedback	5982 (15 studies did not report)	Before after studies, no improvements reported	Low
Mukadam N, 2015 [31]	Dementia	13	5 [0] Denmark (1) UK (1) USA (2) Germany (1) France (1)	GPs and primary care clinics	1) Behaviour, performance or practice - dementia detection and adherence to guidelines 2) Knowledge 3) Attitude	One-to-one (4), Group setting (5), Written information (3), Training to use screening tools (2), Decision support (4), Patient education (1), Specialist consultation (1)	1) Questionnaire: Patient reported healthcare outcomes 2) Knowledge questionnaire: healthcare worker 3) review of medical record notes	1,312 participants and 9 clinics	Cluster RCT (5), controlled before after (1): Improved healthcare outcomes when intervention included both education and structured care management	High
Piot MA, 2021 [32]	Mental health	11 in meta-analysis	Not reported	Nurses	Nurses skills, attitudes and behaviours and mental health outcomes	Simulation: Simulated patient (55) Role-play (40) Virtual reality (12) Manikin (10) Voice simulation (9)	Pre-post tests: Satisfaction (4) Attitudes (88) Skills (25) Knowledge (43) Behaviours (20) Mental health outcomes (7)	Not reported	Randomized and non-randomized controlled studies and single group pre/post studies. Attitude: simulation and inactive control - immediately post test (0.22; 95% CI [0.06; 0.38]), 2–4 month follow up: (0.60; [0.15; 1.0) Skills: simulation and active control − (1.12; [0.39; 1.86]),	High
Rotenberg S, 2022 [11]	All disabilities	78	Reported by region	Medics (37) Nurses (17) Allied Health Professionals (31) Dentists (7) Psychologists (4) Personal Care Workers (5) Community Healthcare workers (2) Pharmacists (2)	Knowledge (57) Competence (42) Attitudes (31) Knowledge (57) Competence (42) Attitudes (31) Confidence (24) Comfort (15) Communication skills (12) Self-Efficacy (11)	Lecture/didactic methods (65) Case study (28) Clinical encounter (26) Placements, experiential, and community-based learning (25) Simulation (24) People with disabilities as a teacher (19)	70% of studies designed their own instruments	Not reported	Narrative review: Use of multiple teaching methods and multi-pronged approaches that emphasise mainstreaming disability in health curricula, sustained approaches that promote systemic change	High
van der Meer L, 2016 [33]	Intellectual disabilities	22	Not reported	Staff’	1) Develop communication plans 2) Improve interaction skills 3) Implement intervention plans 4) Use augmented communication	Presentation/instruction/manual, discussion, modelling/demonstration, role play/practice, video analysis/examples, feedback, self-monitoring/examination	1) Change in behaviour and knowledge/belief of staff, 2) Change in outcomes of people with intellectual disabilities (communication and behaviour)	432 participants	Systematic review: 14 studies provided emerging evidence, with one study proviA9:P10ding conclusive evidence: (number choice opportunities provided increased)	Medium

A broad variety of evaluation methods were used, such as pre- and post-test knowledge assessments, questionnaires at baseline up to 18 months, and immediate post-training evaluations. The impact of the education was typically measured by healthcare worker reports of comfort and attitudes towards people with disabilities, as well as communication skills, knowledge, and self-efficacy. Target outcomes for the training interventions varied, with some focusing on perspective/awareness/comfort, medical and clinical knowledge. Several had unclear outcomes. However, only a few studies considered the longer-term impact, specifically three months post-intervention. The training effect estimates are almost exclusively for high income countries.

### Interviews with international experts

International experts reported that people with disabilities play an important role in improving the quality of care for themselves and others. Interviewees identified several ways that this role would occur, as people with disabilities can: offer unique insights into their experiences and perspectives, identify areas for improvement in their care, serve as advocates and role models, and help to promote a culture of inclusion and understanding in the healthcare system. They can also help to ensure that training programs are relevant, effective, and responsive to the needs of people with disabilities.

Experts reported that good practice examples of training methods included contextualized story-telling and activities adapted by the trainer to the local context. Participants believed that these methods were most effective in engaging learners and improving their knowledge, attitudes, and skills related to disability. However, the success of these methods depended on the quality of the training materials, the experience and skills of the trainers, and the level of buy-in and engagement from learners.

Interviewees identified challenges in providing disability education within healthcare training, citing issues such as lack of standardization in curricula and limited time and resources. They expressed concern that these factors may contribute to inadequate understanding of disability issues among healthcare providers, resulting in disparities in care and outcomes for people with disabilities. Additionally, participants discussed emerging trends in healthcare, such as the use of telemedicine and wearable devices, which they believed would require healthcare workers to develop an even deeper understanding of the needs of people with disabilities. They identified opportunities for scaling training, including integrating disability education into undergraduate degrees, continuing education programs, mentoring, and coaching. Overall, participants viewed the future of disability education for healthcare workers as an exciting opportunity for growth and advancement in the healthcare industry.

### Interviews in Uganda

Overall, the importance of improving communication, understanding, and collaboration between healthcare providers and people with disabilities to promote equitable and high-quality healthcare services was emphasised in interviews with both people with disabilities and healthcare workers.

People with disabilities highlighted that they expected healthcare workers to recognise and be made aware of the challenges they face when trying to access healthcare services. Additionally, they emphasised that they desired to be treated with the same respect and dignity as any other patient, and they value a positive attitude from healthcare providers towards their care.

“We want healthcare workers to know that all people are equal, including those of us with disabilities, and they should treat each person with dignity.” (Male, visual impairment).

“The doctors in the hospital should know that we too are humans, and have blood like them. The difference maybe is that one of my parts is weak, but it doesn’t stop us from getting sick like them.” (Female, physical impairment).

Many of the participants with disabilities acknowledged that better communication skills from healthcare providers, such as clear and concise explanations, are essential to building trust and rapport with people with disabilities.

*“What I know is that for my life to continue being good, it is important for me to be able to visit a healthcare worker who understands my situation and I am not insulted…The care and respect you give me when you see me, and how you speak, is helpful.”* (Male, Albinism).

Similarly, the healthcare workers expressed a need for more training and education on disability to provide high-quality care. The reported training needs ranged from basic training orientation on disability, including communication skills and knowledge on how to navigate the time needed for disability-inclusive care, to how to examine and treat people with disabilities during routine health visits.

*“First of all, we need training because people with disabilities cannot be managed like other individuals. We need training on the forms of disabilities because the different types of disabilities call for different management. So, we can be empowered…and we can manage the expected and non-expected challenges.”(Male, Medical officer)*.

*“We need to know, how do you assess, and how do you support and counsel them. If you do not have those skills sometimes you can mishandle them. For example, you may just look at the cough they have but behind the cough there could be other things.”* (Female, Senior nursing officer).

Furthermore, healthcare providers pointed out the need for adequate information about specialised service needs, how to make referrals and contextual considerations (e.g. cultural, social, economic) to ensure that people with disabilities received appropriate care and were referred for further management.

*“If I recognise the need for specialised care, I would simply write a referral note. However, many times I am unsure of where to refer them, so I am unable to follow up on whether they received the necessary assistance. Writing a referral is the best I can do.”* (Male, Senior medical officer).

*“We can train healthcare workers within the facility on the best practices for interacting with people with disabilities. Even those of us who support them in the community can be trained on how to connect them with specialised services*.” (Male, VHT coordinator).

*“It may be beneficial to collaborate with others who work with individuals with disabilities and provide holistic care. Since we operate at different levels and some hospitals have specialised clinics for individuals with disabilities, working together can help ensure they receive proper services”.* (Female, Midwife)

Involving people with disabilities in training was regarded as a way of facilitating mutual understanding and enabling healthcare workers to better address the specific needs of persons with disabilities, while establishing a designated person for follow-up could enhance continuity of care.

*“Sometimes we are left behind, but if we are invited to health workshops, we can share our experiences with healthcare workers, including how we feel and how we should be treated. We can discuss the specific disabilities we face and the challenges we encounter. This would provide an opportunity for healthcare workers to better understand and appreciate what individuals with disabilities go through.*” (Male, Person with disability councillor, Visual impairment).

The findings suggest that healthcare workers often feel uncertain about referring people with disabilities for specialised care, leading to challenges in ensuring necessary and appropriate healthcare. Training healthcare workers on best practices for interacting with people with disabilities, both within the facility and in the community was recommended to help improve their ability to connect patients with specialised services and provide holistic care.

### Themes and recommendations from the participatory workshop

Five themes for healthcare worker training on disability were generated by consensus from discussions during the participatory workshop. These themes included the need for a focus on:

1. Responsibilities and rights: emphasising understanding of the rights of people with disabilities;
2. Communication: highlighting effective communication for building rapport;
3. Informed consent: focusing on respecting privacy during examinations;
4. Accommodation: advocating for disability-inclusive practices; and
5. Referral and connection: ensuring appropriate referrals and connections to other healthcare services.

The workshop also emphasised the importance of active participation, clear communication, and inclusivity in the design process.

It was recommended that the disability training program should adopt a modular approach, focusing on different aspects of providing healthcare to people with disabilities. It should also focus on increasing knowledge and understanding of disabilities, and improving the attitudes and practices of healthcare workers towards people with disabilities. Emphasis should be placed on adopting a disability-inclusive approach, training healthcare workers on the social model of disability, recognising the impact of societal barriers on the lives of people with disabilities, and addressing these barriers to promote greater inclusion and participation in society. Cultural sensitivity and the use of appropriate terminology are essential help to ensure that the training is inclusive and relevant to the diverse population of people with disabilities that healthcare workers may encounter.

Interaction with people with disabilities was recommended as a key component. This will involve inviting people with disabilities to participate in the training sessions to share their experiences and perspectives. The training should also adopt a learner-centred and participatory approach, based on the values of the healthcare worker, promoting reflection on their own values and beliefs and applying them in their practice, which may be effective in promoting a sense of ownership and commitment to working with people with disabilities.

Ongoing mentorship and peer learning should support the training, pairing healthcare workers with experienced mentors to provide guidance and support as they apply their new knowledge and skills in practice. Practical skills should be included in the training, such as techniques for measuring the weight of a person with a physical impairment, to ensure comprehensive and considerate examination and treatment of people with disabilities.

### Training material structure

**Fig. 2:**
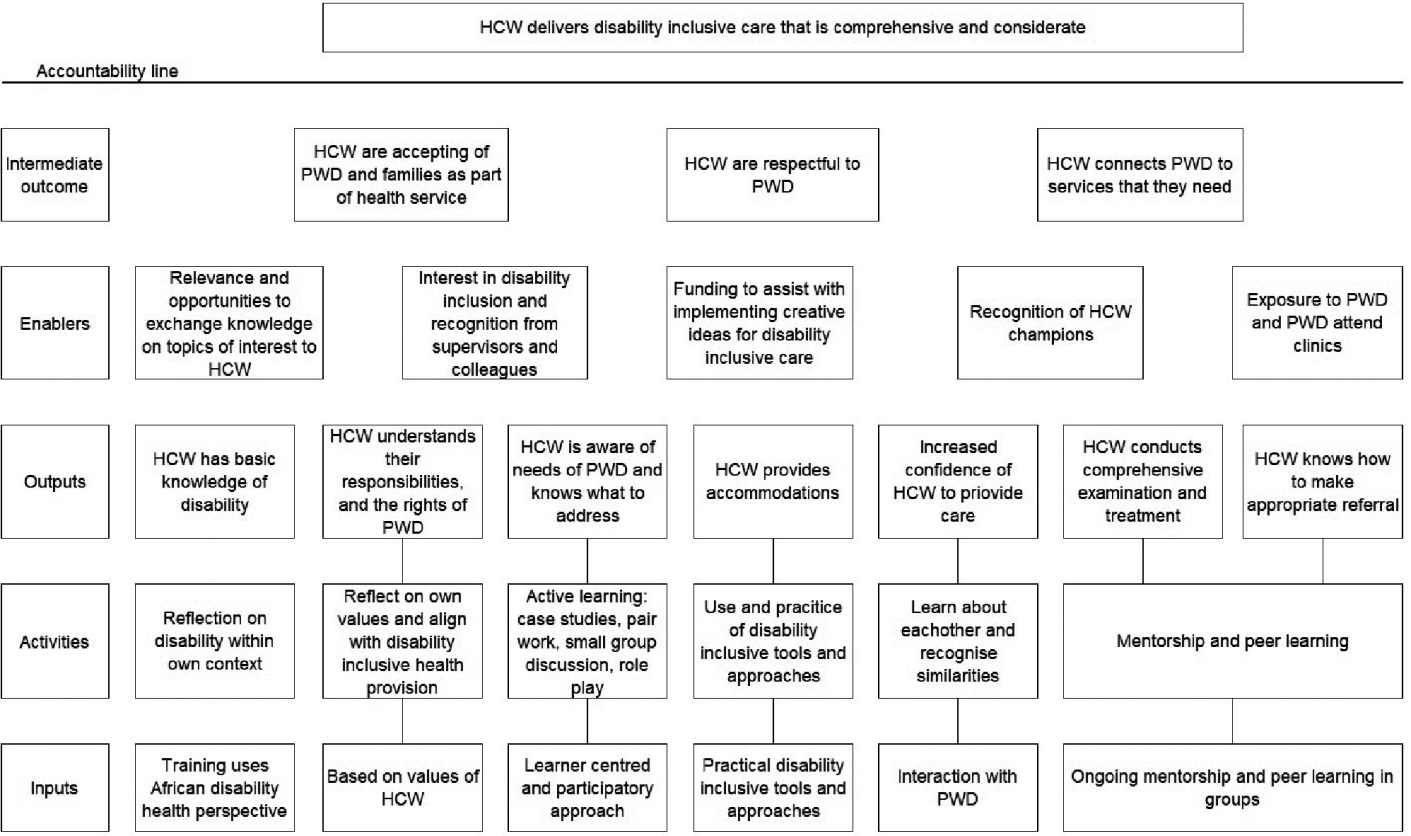
Theory of change *HCW = healthcare worker, PWD = people with disabilities

The theory of change provided a clear and logical structure for the development of the training framework and helped to ensure that the framework was comprehensive, evidence-based and responsive to the specific needs and experiences of people with disabilities in Uganda. The desired outcomes of the training included increasing the knowledge, skills and attitudes of healthcare workers on disability, to ensure that people with disabilities are expected, accepted and connected within health systems. The ceiling of responsibility is the distal outcome that healthcare workers provide disability inclusive care that is considerate and comprehensive. Key components and activities that would be necessary to achieve the proximal outcomes included interaction with people with disabilities, a learner centred participatory approach based on the values of healthcare workers, ongoing mentorship and peer learning (Fig. 2).

The developed training programme included a range of activities such as pre-training self-assessments, interactive workshops, case studies and mentoring. The training framework also included specific provisions for accessibility and inclusion, recognising the importance of addressing the specific needs and experiences of people with disabilities.

The training will be delivered by a healthcare worker and a person with a disability. They will facilitate the training in person over one day for various healthcare workers (nurses, technicians, community health workers, allied health professionals, doctors). The programme will focus on key areas such understanding disability, good practices in work, and personal motivations for providing disability-inclusive healthcare. Participants will learn about routine health needs, communication skills, assessing and treating persons with disabilities, appropriate referrals, and ensuring accessibility in healthcare settings.

The training program aims to achieve its goals through several strategies. It promotes a disability-inclusive approach by engaging people with disabilities and encouraging active participation and reflection. Ongoing mentorship and peer learning opportunities are provided for continuous support. Practical tools are shared to equip healthcare workers with necessary skills. Cultural sensitivity is fostered to ensure healthcare workers can provide care that is respectful and responsive to diverse cultural backgrounds and language preferences. By incorporating these strategies, the training program aims to empower healthcare workers with the expertise needed to provide inclusive and effective healthcare services for persons with disabilities.

## Discussion

Developing a comprehensive training programme for healthcare workers on disability is an important step in addressing barriers to healthcare access for people with disabilities [3]. We used the Medical Research Council approach to develop the training material [17], considering practical solutions to improve the knowledge and skills of healthcare workers on disability. Our umbrella literature search revealed that lectures and didactic methods were commonly used in disability education, often combined with other approaches such as case studies and simulations. The review also highlighted the importance of using various teaching methods and including people with disabilities in disability education. The impact of education was measured in various ways, including through healthcare worker reports on comfort, attitudes, communication skills, knowledge, and self-efficacy. There is need for a standardised approach to allow comparison between contexts and countries. Standardisation in curricula and limited time and resources were identified as challenges in providing disability education within healthcare training.

People with disabilities and healthcare workers in Uganda expressed the need for better communication skills from healthcare providers, training on disability, and recognition of challenges faced by people with disabilities. These findings are echoed in other studies globally [19–22]. The participatory workshop successfully generated a comprehensive and inclusive framework for a disability training programme, incorporating the diverse perspectives of people with disabilities and healthcare workers. Our findings are consistent with other studies that highlight the importance of disability education in healthcare training, utilising various teaching methods and incorporating the perspectives of people with disabilities [23, 24]. The next step is to pilot-test the training programme with healthcare workers in Luuka District, Uganda. During pilot testing, the training program will also consider the cultural context in which it will be delivered, as previous research has shown that cultural competence is essential for providing effective and appropriate care to people with disabilities [25, 26].

One of the strengths of our study was the use of the MRC approach to develop the training material, which enabled us to systematically examine training needs and develop practical solutions to improve the knowledge and skills of healthcare workers on disability. In addition, the study encompasses a diverse range of health worker cadres and people with varying types of disabilities, ensuring a comprehensive exploration of perspectives and experiences. Our study also has limitations to consider when interpreting the results. A systematic umbrella review was undertaken rather than a systematic review. Recruitment through existing networks, NGOs, and health facilities may not capture the perspectives of people with disabilities who are not affiliated with these organisations. We included the perspectives of people with disabilities and healthcare workers in Uganda, and thus the generalisability of the disability training to other contexts may be limited. While we did include the opinions of international experts and a global umbrella review, further research is needed to confirm the effectiveness and applicability of the training program in different settings, as the umbrella review noted important gaps in the literature.

Our findings have important implications for policy, programmes and research. Specifically, our study suggests that a disability training programme should be inclusive of diverse cultural backgrounds and adaptable to specific needs, and should incorporate the perspectives of both people with disabilities and healthcare workers. These findings could be used to inform the adaptation of future training programs for healthcare workers in Uganda and other resource-limited settings. Nevertheless, people with disabilities are a diverse group with varying needs and this has implications for training. While advocating for overall disability training, it is essential to also focus on different types of impairment. Additionally, the needs of different healthcare workers will vary due to their diverse roles, raising questions about the feasibility of a generic training programme. Policy support is vital to ensure implementation and support of disability training for healthcare workers (e.g. mandating inclusion in medical and nursing curricula). Future studies could use more objective measures of impact, such as patient outcomes or changes in healthcare service provision.

## Conclusion

The proposed development of a disability training for healthcare workers aims to address the barriers and challenges faced by people with disabilities in accessing health care services in Africa. The training will adopt a modular approach. Components of the training include interaction with people with disabilities, a learner-centred and participatory approach, ongoing mentorship and peer learning, practical tools to deliver a comprehensive and considerate examination and treatment, and cultural sensitivity.

### Electronic supplementary material

Below is the link to the electronic supplementary material.

Supplementary Material 1

## Data Availability

The majority of the datasets supporting the conclusions of this article are included within the article and its additional files. However, underlying interview data associated with this study will not be made freely available, as the small number of healthcare worker and people with disabilities makes data potentially identifying. Applications for access to the raw qualitative data for this study should be made via email to the corresponding author tracey.smythe@lshtm.ac.uk, outlining the purpose of the proposed analyses and the data requested. These applications will be reviewed by the LSHTM’s data access committee, and if accepted, the requested data will be shared.
