# Correction: Co-development of a training programme on disability for healthcare workers in Uganda

**DOI:** 10.1186/s12913-024-11004-0

**Published:** 2024-05-16

**Authors:** Tracey Smythe, Andrew Sentoogo Ssemata, Sande Slivesteri, Femke Bannink Mbazzi, Hannah Kuper

**Affiliations:** 1 International Centre for Evidence in Disability, London School of Hygiene & Tropical Medicine, London, UK; 2 Division of Physiotherapy, Department of Health and Rehabilitation Sciences, Stellenbosch University, Cape Town, South Africa; 3 Medical Research Council/Uganda Virus Research Institute and London School of Hygiene & Tropical Medicine Uganda Research Unit, Entebbe, Uganda; 4 Department of Global Health and Development, London School of Hygiene & Tropical Medicine, London, UK

**Correction:** **BMC Health Serv Res 24, 418 (2024)**

**https://doi.org/10.1186/s12913-024-10918-z**

In Table 2 of this article, multiple errors were present:

- The citations to the references (in between square brackets) were missing in the first column and the corresponding references were missing from the reference list.
- In columns 4-6/row 2, some of the numbers in between brackets were incorrectly formatted as reference citations (as a result of a typesetting mistake); formatted in bold in the incorrect version of Table 2 below.
- In column 7/row 5, a number in between brackets was incorrectly formatted as reference citations (as a result of a typesetting mistake); formatted in bold in the incorrect version of Table 2 below.

In addition, some of the author correction instructions were published as article notes (as a result of a typesetting mistake).

The incorrect and correct version of Table 2 can be found below, as well as a list of the missing references in the reference list. The article has been updated to correct the errors in Table 2, to add the missing references in the reference list and to remove the invalid article notes. The publisher apologises to the authors and readers for the inconvenience caused by the errors.

**List of missing references:**

11. Rotenberg S, Rodríguez Gatta D, Wahedi A, Loo R, McFadden E, Ryan S. Disability training for health workers: A global evidence synthesis. Disabil Health J. 2022;15(2):101260.

27. Adirim Z, Sockalingam S, Thakur A. Post-graduate Medical Training in Intellectual and Developmental Disabilities: a Systematic Review. Acad Psychiatry. 2021;45(3):371–81.

28. Booth A, Scantlebury A, Hughes-Morley A, Mitchell N, Wright K, Scott W, et al. Mental health training programmes for non-mental health trained professionals coming into contact with people with mental ill health: a systematic review of effectiveness. BMC Psychiatry. 2017;17(1):196.

29. Cox A, Dube C, Temple B. The influence of staff training on challenging behaviour in individuals with intellectual disability: a review. J Intellect Disabil. 2015;19(1):69–82.

30. Ioerger M, Flanders R, French-Lawyer J, Turk M. Interventions to Teach Medical Students About Disability: A Systematic Search and Review. Am J Phys Med Rehabil. 2019;98(7):577–99.

31. Mukadam N, Cooper C, Kherani N, Livingston G. A systematic review of interventions to detect dementia or cognitive impairment. Int J Geriatr Psychiatry. 2015;30(1):32–45.

32. Piot M, Dechartres A, Attoe C, Romeo M, Jollant F, Billon G, et al. Effectiveness of simulation in psychiatry for nursing students, nurses and nurse practitioners: A systematic review and meta-analysis. J Adv Nurs. 2022;78(2):332–47.

33. van der Meer L, Matthews T, Ogilvie E, Berry A, Waddington H, Balandin S, et al. Training Direct-Care Staff to Provide Communication Intervention to Adults With Intellectual Disability: A Systematic Review. Am J Speech Lang Pathol. 2017;26(4):1279–95.

**Incorrect Table** 2** and Correct Table **2** (respectively):**

**Table 2:** Training on disability effect estimates for healthcare workers reported in systematic reviews (2015–2022)

Primary author, Year [Ref]	Target disability	Studies N	Countries [LMICs] Country name	Target cadre (n)	Target outcome (n)	Pedagogical methodology (n)	Measurement tools/ Evaluation methodology (n)	Participants trained N	Evidence of impact	Confidence rating
Adirim, 2021 [1]	Intellectual and developmental disabilities	16	2 [0] USA **[14]** Canada (2)	Pediatrics **[12]** Psychiatry (3) Family Medicine (1)	Awareness **[6]** Knowledge (13) Other/unclear (1)	Clinical rotation: (9/16) theoretical/ pedagogical: (14/16) didactic or seminar: (12/14) clinical practice; (10/11), simulation, (1/11). Immersive experiences: (7/16) interactive approaches (5/16)	Knowledge assessment (9/16) Self-reported learning: (9/16) Evaluation of intervention (3/16) Clinical changes (2/16) Observed behavioural change (1)	Not reported	Narrative summary: improved knowledge, skills, competence, positive attitudes	Low
Booth A, 2017	Mental health	2	2 [0] USA (2)	a) Child welfare case officers b) Community practitioners	Knowledge and referral	Face-to-face, didactic and interactive training with video demonstrations of available evidence based practice	54 item questionnaire developed by study team to evaluate knowledge of mental health conditions	a) 67 b)182	Intervention group had significantly increased awareness of evidence based practice	Medium
Cox AD, 2015	Intellectual disabilities	19	Not reported	Direct care, psychologist, manager not reported (8)	1) Health outcome: Improvement in client behaviour: (9/19)	Not reported	1) Direct observation of client behaviour (8) (Combination of two study groups) 2) questionnaires (9)	Not reported	Narrative summary: trend toward change in behaviour of clients	Low
Ioerger M, 2019	All disabilities	77	12 [4] USA (36) UK (19) Canada (6) Australia (7) Brazil (1) Nigeria (1) Pakistan (1) S.Africa (2) Croatia (1) Ireland (1) Israel (1) New Zealand (1)	Medical students	Students' disability knowledge (35), skills (28), or attitudes (35)	Lectures most common (36), followed by reflection **[25]** and small group discussions (25)	1) Attitude change 2) Skills acquired 3) Knowledge 4) General feedback	5982 (15 studies did not report)	Before after studies, no improvements reported	Low
Mukadam N, 2015	Dementia	13	5 [0] Denmark (1) UK (1) USA (2) Germany (1) France (1)	GPs and primary care clinics	1) Behaviour, performance or practice—dementia detection and adherence to guidelines 2) Knowledge 3) Attitude	One-to-one (4), Group setting (5), Written information (3), Training to use screening tools (2), Decision support (4), Patient education (1), Specialist consultation (1)	1) Questionnaire: Patient reported healthcare outcomes 2) Knowledge questionnaire: healthcare worker 3) review of medical record notes	1,312 participants and 9 clinics	Cluster RCT (5), controlled before after (1): Improved healthcare outcomes when intervention included both education and structured care management	High
Piot MA, 2021	Mental health	11 in meta-analysis	Not reported	Nurses	Nurses skills, attitudes and behaviours and mental health outcomes	Simulation: Simulated patient (55) Role-play (40) Virtual reality (12) Manikin (10) Voice simulation (9)	Pre-post tests: Satisfaction (4) Attitudes (88) Skills (25) Knowledge (43) Behaviours (20) Mental health outcomes (7)	Not reported	Randomized and non-randomized controlled studies and single group pre/post studies. Attitude: simulation and inactive control—immediately post test (0.22; 95% CI [0.06; 0.38]), 2–4 month follow up:(0.60; [0.15; 1.0) Skills: simulation and active control—(1.12; [0.39; 1.86]),	High
Rotenberg S, 2022	All disabilities	78	Reported by region	Medics (37) Nurses (17) Allied Health Professionals (31) Dentists (7) Psychologists (4) Personal Care Workers (5) Community Healthcare workers (2) Pharmacists (2)	Knowledge (57) Competence (42) Attitudes (31) Knowledge (57) Competence (42) Attitudes (31) Confidence (24) Comfort (15) Communication skills (12) Self-Efficacy (11)	Lecture/didactic methods (65) Case study (28) Clinical encounter (26) Placements, experiential, and community-based learning (25) Simulation (24) People with disabilities as a teacher (19)	70% of studies designed their own instruments	Not reported	Narrative review: Use of multiple teaching methods and multi-pronged approaches that emphasise mainstreaming disability in health curricula, sustained approaches that promote systemic change	High
van der Meer L, 2016	Intellectual disabilities	22	Not reported	Staff'	1) Develop communication plans 2) Improve interaction skills 3) Implement intervention plans 4) Use augmented communication	Presentation/instruction/manual, discussion, modelling/demonstration, role play/practice, video analysis/examples, feedback, self-monitoring/examination	1) Change in behaviour and knowledge/belief of staff, 2) Change in outcomes of people with intellectual disabilities (communication and behaviour)	432 participants	Systematic review: 14 studies provided emerging evidence, with one study proviA9:P10ding conclusive evidence: (number choice opportunities provided increased)	Medium

**Table 2:** Training on disability effect estimates for healthcare workers reported in systematic reviews (2015–2022)

Primary author, Year [Ref]	Target disability	Studies N	Countries [LMICs] Country name	Target cadre (n)	Target outcome (n)	Pedagogical methodology (n)	Measurement tools/ Evaluation methodology (n)	Participants trained N	Evidence of impact	Confidence rating
Adirim Z, 2021 [27]	Intellectual and developmental disabilities	16	2 [0] USA (14) Canada (2)	Pediatrics (12) Psychiatry (3) Family Medicine (1)	Awareness (6) Knowledge (13) Other/unclear (1)	Clinical rotation: (9/16) theoretical/ pedagogical: (14/16) didactic or seminar: (12/14) clinical practice; (10/11), simulation, (1/11). Immersive experiences: (7/16) interactive approaches (5/16)	Knowledge assessment (9/16) Self-reported learning: (9/16) Evaluation of intervention (3/16) Clinical changes (2/16) Observed behavioural change (1)	Not reported	Narrative summary: improved knowledge, skills, competence, positive attitudes	Low
Booth A, 2017 [28]	Mental health	2	2 [0] USA (2)	a) Child welfare case officers b) Community practitioners	Knowledge and referral	Face-to-face, didactic and interactive training with video demonstrations of available evidence based practice	54 item questionnaire developed by study team to evaluate knowledge of mental health conditions	a) 67 b)182	Intervention group had significantly increased awareness of evidence based practice	Medium
Cox A, 2015 [29]	Intellectual disabilities	19	Not reported	Direct care, psychologist, manager not reported (8)	1) Health outcome: Improvement in client behaviour: (9/19)	Not reported	1) Direct observation of client behaviour (8) (Combination of two study groups) 2) questionnaires (9)	Not reported	Narrative summary: trend toward change in behaviour of clients	Low
Ioerger M, 2019 [30]	All disabilities	77	12 [4] USA (36) UK (19) Canada (6) Australia (7) Brazil (1) Nigeria (1) Pakistan (1) S.Africa (2) Croatia (1) Ireland (1) Israel (1) New Zealand (1)	Medical students	Students' disability knowledge (35), skills (28), or attitudes (35)	Lectures most common (36), followed by reflection (25) and small group discussions (25)	1) Attitude change 2) Skills acquired 3) Knowledge 4) General feedback	5982 (15 studies did not report)	Before after studies, no improvements reported	Low
Mukadam N, 2015 [31]	Dementia	13	5 [0] Denmark (1) UK (1) USA (2) Germany (1) France (1)	GPs and primary care clinics	1) Behaviour, performance or practice—dementia detection and adherence to guidelines 2) Knowledge 3) Attitude	One-to-one (4), Group setting (5), Written information (3), Training to use screening tools (2), Decision support (4), Patient education (1), Specialist consultation (1)	1) Questionnaire: Patient reported healthcare outcomes 2) Knowledge questionnaire: healthcare worker 3) review of medical record notes	1,312 participants and 9 clinics	Cluster RCT (5), controlled before after (1): Improved healthcare outcomes when intervention included both education and structured care management	High
Piot M, 2021 [32]	Mental health	11 in meta-analysis	Not reported	Nurses	Nurses skills, attitudes and behaviours and mental health outcomes	Simulation: Simulated patient (55) Role-play (40) Virtual reality (12) Manikin (10) Voice simulation (9)	Pre-post tests: Satisfaction (4) Attitudes (88) Skills (25) Knowledge (43) Behaviours (20) Mental health outcomes (7)	Not reported	Randomized and non-randomized controlled studies and single group pre/post studies. Attitude: simulation and inactive control—immediately post test (0.22; 95% CI [0.06; 0.38]), 2–4 month follow up:(0.60; [0.15; 1.0) Skills: simulation and active control—(1.12; [0.39; 1.86]),	High
Rotenberg S, 2022 [11]	All disabilities	78	Reported by region	Medics (37) Nurses (17) Allied Health Professionals (31) Dentists (7) Psychologists (4) Personal Care Workers (5) Community Healthcare workers (2) Pharmacists (2)	Knowledge (57) Competence (42) Attitudes (31) Knowledge (57) Competence (42) Attitudes (31) Confidence (24) Comfort (15) Communication skills (12) Self-Efficacy (11)	Lecture/didactic methods (65) Case study (28) Clinical encounter (26) Placements, experiential, and community-based learning (25) Simulation (24) People with disabilities as a teacher (19)	70% of studies designed their own instruments	Not reported	Narrative review: Use of multiple teaching methods and multi-pronged approaches that emphasise mainstreaming disability in health curricula, sustained approaches that promote systemic change	High
van der Meer L, 2016 [33]	Intellectual disabilities	22	Not reported	Staff'	1) Develop communication plans 2) Improve interaction skills 3) Implement intervention plans 4) Use augmented communication	Presentation/instruction/manual, discussion, modelling/demonstration, role play/practice, video analysis/examples, feedback, self-monitoring/examination	1) Change in behaviour and knowledge/belief of staff, 2) Change in outcomes of people with intellectual disabilities (communication and behaviour)	432 participants	Systematic review: 14 studies provided emerging evidence, with one study proviA9:P10ding conclusive evidence: (number choice opportunities provided increased)	Medium

