# Supplementary material for: Co-development of a training programme on disability for healthcare workers in Uganda
Source: BMC Health Serv Res. 2024 Apr 3;24:418. doi: 10.1186/s12913-024-10918-z (PMC10988913; doi:10.1186/s12913-024-10918-z)
Supplement: Supplementary file 1 — Supplementary Material 1 [file 12913_2024_10918_MOESM1_ESM.docx]

**Supplementary file 1: Topic guide for interview with global stakeholders**

Please can you explain the training programmes that you have been involved in.

Please could you outline how these programmes were created.

What has worked well with your training programme?

Are there any other strengths?

What is the aim of the training programme, how do you measure outcome, how do you know if you have succeeded?

What has been difficult in the set up or roll out of the training programme?

What would you do differently next time?

Any other suggestions of who to speak with or documents/training to read?

**Supplementary file 2: Participant demographics***

|  |  | **Person with disabilities (N=27)** | **Health workers (N=17)** |
| --- | --- | --- | --- |
|  | **Characteristic** | **n (%)** | **n (%)** |
| Sex |  |  |  |
|  | Female | 15 (56) | 9 (53) |
|  | Male | 12 (44) | 8 (47) |
| Impairment | Cognitive | 5 (19) | - |
|  | Hearing | 6 (21) | - |
|  | Multiple | 5 (19) | - |
|  | Albinism | 1 (3) | - |
|  | Physical | 5 (19) | - |
|  | Visual | 5 (19) | - |
| Age | Median age (IQR) | 26 (21-40) years | 34 (22 – 55) years |
| Occupation | Formal employment | 8 (30) | - |
|  | Informal employment | 15 (56) | - |
|  | Unemployed/ students | 4 (14) | - |
|  | Medical Clinical officer | - | 6 (35) |
|  | Health officer | - | 2 (12) |
|  | Laboratory Technician | - | 2 (12) |
|  | Midwife | - | 2 (12) |
|  | Nursing officer | - | 3 (17) |
|  | Rehabilitation officer | - | 2 (12) |

*semi-structured interviews undertaken in Uganda

**Supplementary file 3: Healthcare worker interview topic guide**

**Objective:** To gather information to help design training programme for healthcare workers about disability

### These questions should be used to guide discussion but do not have to be used in the sequence listed below. The interviewer should follow up on any additional issues that may arise and seem important in relation to the issues above.

### **Introduction**

### Greet them and thank them for their time

### Identify yourself by name and organisation.

### Read out the information sheet. Remind them of confidentiality and anonymity. Check if they have any questions. Remind them that they are free to decline to answer any of the questions or stop the interview at any time.

### Record their consent/assent in the relevant form OR record verbal consent.

### Start recording

### **Notes:** the following details must be recorded in field notes

| Participant Code |  |
| --- | --- |
| Interview date and time |  |
| Interview location or mode (phone, video) |  |
| Interviewer |  |
| Interviewee(Record caregiver name if they are present) |  |
| Gender |  |
| Age |  |
| General observations (anything which might impact how the interview is conducted) |  |

### **Healthcare worker background**

1. Can you please describe to me your tell me more about your role as [job title].

**Prompt:** What activities do you do in this role?

1. As we have discussed, we are gathering information to help design training programme for healthcare workers about disability. How would you define disability?
2. In your job, how often do you treat or provide services to people with disabilities?

***Provision of services***

1. Can you describe 2-3 recent examples to me of when you have provided services to people with disabilities?

**Prompt**

- Impairment type
- Healthcare need/service need
- Challenges in providing care
- Supporting factors in providing care

1. What do you think are the main challenges that people with disabilities experience when seeking healthcare in the facility where you work?

**Prompts,** if needed

- Accessibility
- Cost of service
- Attitudes of staff
- Availability of services needed
- Difficulties in communication
- Specialist care (e.g. diabetes, treatment for heart conditions) or referral to specialist care

1. What do you think could help in overcoming these challenges?

**Prompt:** what might help you?

- How do colleagues support you in providing health care to people with disabilities? (eg any specialised services or nurses who are specifically trained?)
- What might help people with disabilities?
- What might help the facility and organisation?

1. In general, how confident do you feel in providing services to people with disabilities?

***Training on disability***

1. Did you receive any training about disability? If so, please can you describe the training that you received.
2. Do you think that training on disability would be helpful? If so, can you describe what you think would be most helpful

**Prompt**

- Topics to include: e.g. attitudes, methods for working with people with disabilities
- Mode of teaching: small groups, lectures, learning from people with disabilities themselves
- Location
- Duration
- Timing (during training or on job training)
- By whom

1. What would be motivation for healthcare workers to attend training on disability?

**Prompt**

- Continuing professional development points
- Increased ability to do their job well
- Increased respect and social standing within work or community
- Interest in working with people with disabilities
- Increased chances of promotion
- Personal experience with people with disabilities outside of my job

1. Other than training, is there anything else that might be helpful for healthcare workers to know how to properly treat and refer people with disabilities?
2. Is there anything else you would like to add?

**Supplementary file 4: Person with disability interview topic guide**

### Objective: To find out about people’s experience accessing healthcare, common issues, and advice on establishment of participatory groups

### These questions should be used to guide discussion but do not have to be used in the sequence listed below. The interviewer should follow up on any additional issues that may arise and seem important in relation to the issues above.

### Introduction

### - Greet them and thank them for their time

### - Identify yourself by name and organisation.

### - Read out the information sheet. Remind them of confidentiality and anonymity. Check if they have any questions. Remind them that they are free to decline to answer any of the questions or stop the interview at any time.

### - Record their consent/assent in the relevant form OR record verbal consent.

### - Start recording

| ***Table 1. To be completed for each participant*** | |
| --- | --- |
| *Participant Code* |  |
| *Interview date and time* |  |
| *Interview location or mode (phone, video)* |  |
| *Interviewer* |  |
| *Interviewee*  *(Record caregiver name if they are present)* |  |
| *Nature of impairment(s)* |  |
| *Gender* |  |
| *Age* |  |
| *General observations (anything which might impact how the interview is conducted)* |  |

**Background**

1. Could you tell me about your disability (or health difficulties) please?

**Prompts:**

- How would you describe your disability? For example, what are the difficulties you experience in day to day life?
- Do you use any assistive devices to help you?
  1. If no, would you benefit from these, or do you have access to these?
- Who helps you with daily activities if needed?
- Please describe your household situation. Who do you live with?

**Experience accessing general healthcare**

1. Can you tell me about the last time that you were unwell?

**Prompts:**

- Can you describe to me how you felt? What was the matter?
- What did you do try to make it a bit better?
- [If the person describes seeking healthcare, move on to question 3]
- [If the person did not describe seeking healthcare]:
  - Could you tell me more about why you decided not to go to a clinic or hospital?
  - [If the person didn’t seek care]: Can you tell me about the last time that you went to a clinic or hospital?

1. [If person sought care]: Can you tell me about this time that you went to a clinic or hospital?

**Prompts**

- What made you decide to go?
- How did you get there? What did that involve?
- Interaction in facility: Did you get the care that you wanted or needed?
- What was the best and worst aspects of the experience?

1. Have you received any services from a Village Healthcare worker or Community Healthcare worker?

**Prompts**

- What service did you receive?

Now I would like to ask you some questions about your experiences in receiving health services. For these, please think back to the last few times you’ve received these services

1. In general, are you able to get healthcare when you need it? Can you explain your answer?
2. What do you think could be done to improve your experience of health care?

**Prompts:**

- Awareness

- Accessibility

- Affordability

- Attitudes

**Supplementary File 5:Summary of findings from the quality assessment of selected studies using AMSTAR2**

| Authors, Year of publication [Reference] | Q1 | Q2 | Q3 | Q4 | Q5 | Q6 | Q7 | Q8 | Q9 | Q10 | Q11 | Q12 | Q13 | Q14 | Q15 | Q16 | Overall confidence Rating |
| --- | --- | --- | --- | --- | --- | --- | --- | --- | --- | --- | --- | --- | --- | --- | --- | --- | --- |
| Adirim, 2021 | Yes | No | Yes | Yes | Yes | Yes | No | Yes | Yes | No | NMA | NMA | Yes | Yes | No | Yes | Low |
| Booth A, 2017 | Yes | Yes | Yes | Yes | Yes | Yes | Partially | Yes | Yes | No | NMA | NMA | Yes | Yes | No | Yes | Medium |
| Cox AD, 2015 | Yes | No | Yes | Yes | No | No | No | Yes | No | No | NMA | NMA | Yes | Yes | No | Yes | Low |
| Ioerger M, 2019 | Yes | No | Yes | Yes | No | Yes | No | Yes | Yes | No | NMA | NMA | Yes | Yes | No | Yes | Low |
| Mukadam N, 2015 | Yes | No | Yes | Yes | Yes | Yes | Yes | Yes | Yes | No | NMA | NMA | Yes | Yes | Yes | Yes | High |
| Piot MA, 2021 | Yes | Yes | Yes | Yes | Yes | Yes | No | Yes | Yes | No | Yes | Yes | Yes | Yes | Yes | Yes | High |
| Rotenberg S, 2022 | Yes | Yes | Yes | Yes | Yes | Yes | Partially | Yes | Yes | No | NMA | NMA | Yes | Yes | Yes | Yes | High |
| van der Meer L, 2016 | Yes | No | Yes | YEs | Yes | Yes | No | Yes | Yes | No | NMA | NMA | Yes | Yes | No | Yes | Medium |

NMA: No Meta-Analysis

**AMSTAR@ Questions**: **Q1:** Did the research questions and inclusion criteria for the review include the components of PICO?; **Q2:** Did the report of the review contain an explicit statement that the review methods were established prior to the conduct of the review and did the report justify any significant deviations from the protocol?; **Q3:** Did the review authors explain their selection of the study designs for inclusion in the review?; **Q4:** Did the review authors use a comprehensive literature search strategy?; **Q5:** Did the review authors perform study selection in duplicate?; **Q6:** Did the review authors perform data extraction in duplicate?; **Q7**: Did the review authors provide a list of excluded studies and justify the exclusions?; **Q8:** Did the review authors describe the included studies in adequate detail?; **Q9:** Did the review authors use a satisfactory technique for assessing the risk of bias (RoB) in individual studies that were included in the review?; **Q10:** Did the review authors report on the sources of funding for the studies included in the review?; **Q11:** If meta-analysis was performed did the review authors use appropriate methods for statistical combination of results?; **Q12:** If meta-analysis was performed, did the review authors assess the potential impact of RoB in individual studies on the results of the meta-analysis or other evidence synthesis?; **Q13:** Did the review authors account for RoB in individual studies when interpreting/ discussing the results of the review?; **Q14:** Did the review authors provide a satisfactory explanation for, and discussion of, any heterogeneity observed in the results of the review?; **Q15:** If they performed quantitative synthesis did the review authors carry out an adequate investigation of publication bias (small study bias) and discuss its likely impact on the results of the review?; **Q16:** Did the review authors report any potential sources of conflict of interest, including any funding they received for conducting the review
